# Diagnostic and prognostic value of an ejection fraction adjusted for myocardial remodeling

**DOI:** 10.3389/fcvm.2024.1349338

**Published:** 2024-05-09

**Authors:** Wei Xuan Chan, Amit Kaura, Abdulrahim Mulla, Dimitri Papadimitriou, Benjamin Glampson, Erik Mayer, Anoop S. V. Shah, Jamil Mayet, Choon Hwai Yap

**Affiliations:** ^1^ Department of Bioengineering, Imperial College London, London, United Kingdom; ^2^ National Institute for Health Research Imperial Biomedical Research Centre, Imperial College London and Imperial College Healthcare NHS Trust, London, United Kingdom; ^3^ Department of Surgery & Cancer, Imperial College London, London, United Kingdom; ^4^ Department of Non-Communicable Disease Epidemiology, London School of Hygiene and Tropical Medicine, London, United Kingdom

**Keywords:** heart failure with preserved ejection fraction, mid-wall ejection fraction, cardiac remodelling, left ventricular mass (LVM), echocardiography

## Abstract

**Introduction:**

Ejection fraction (EF) is widely used to evaluate heart function during heart failure (HF) due to its simplicity compared but it may misrepresent cardiac function during ventricular hypertrophy, especially in heart failure with preserved EF (HFpEF). To resolve this shortcoming, we evaluate a correction factor to EF, which is equivalent to computing EF at the mid-wall layer (without the need for mid-layer identification) rather than at the endocardial surface, and thus better complements other complex metrics.

**Method:**

The retrospective cohort data was studied, consisting of 2,752 individuals (56.5% male, age 69.3 ± 16.4 years) admitted with a request of a troponin test and undergoing echocardiography as part of their clinical assessment across three centres. Cox-proportional regression models were constructed to compare the adjusted EF (EFa) to EF in evaluating risk of heart failure admissions.

**Result:**

Comparing HFpEF patients to non-HF cases, there was no significant difference in EF (62.3 ± 7.6% vs. 64.2 ± 6.2%, *p* = 0.79), but there was a significant difference in EFa (56.6 ± 6.4% vs. 61.8 ± 9.9%, *p* = 0.0007). Both low EF and low EFa were associated with a high HF readmission risk. However, in the cohort with a normal EF (EF ≥ 50%), models using EFa were significantly more associative with HF readmissions within 3 years, where the leave one out cross validation ROC analysis showed a 18.6% reduction in errors, and Net Classification Index (NRI) analysis showed that risk increment classification of events increased by 12.2%, while risk decrement classification of non-events decreased by 16.6%.

**Conclusion:**

EFa is associated with HF readmission in patients with a normal EF.

## Graphical Abstract

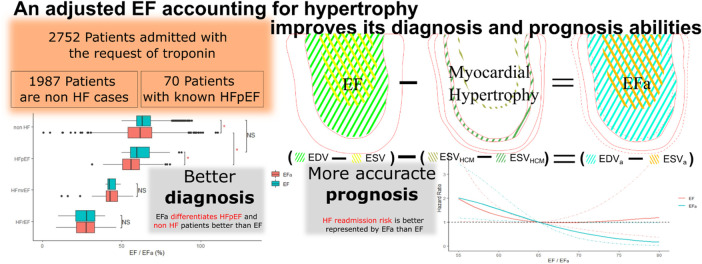

## Highlight

• EF of HFpEF patients are confounded by myocardial geometric changes in disease remodeling.
• Proposed correction to EF resolves this and associate closely with rehospitalization risks.
• A new subgroup of HFpEF patients with reduced contractile function can thus be identified.
• Following, drug and management trials should be assessed accounting for this new subgroup.

## Introduction

Systolic and diastolic Heart failure (HF), a leading cause of morbidity and mortality world-wide, is a complex clinical syndrome reflecting deficient cardiac output. Great progress has been made in the treatment of heart failure where the systolic function is impaired but only recently studies evaluating treatments for those with preserved systolic function have shown some progress using SGLT2 inhibitors (1) and GLP-1 Agonist (2) targeting their respective comorbidities.

The ejection fraction (EF) is widely used for the evaluation of cardiac health and contractile function during HF, as it has been shown to have prognostic capabilities (3). However, studies have highlighted inadequacies of the parameter. For cases of HF with a preserved EF (HFpEF), EF remains in the normal reference range and fails to indicate disease. Further, assessment and treatments of patients where the EF is between 40% to 50%, currently termed as HF mildly reduced EF (HFmrEF), remain uncertain. Also, EF is shown to be modulated by geometric changes to the left ventricle (LV), such as during HF remodeling. Increased wall thickness was shown to enhance EF (4), while dilated LV chamber was shown to reduce EF (5). These modulations make it difficult for EF to be an unbiased representation of cardiac function.

We have recently demonstrated the mechanism for these geometric effects (6). We showed that myocardial deformations at the endocardial surface are naturally enhanced by the inward radial displacements during contractions, and because the extent of the enhancement is modulated by LV geometric characteristics such as wall thickness, endocardial deformations become dependent the geometric shape of the LV as well. Since EF is measured using the endocardial boundary, it is similarly modulated by LV geometric changes, thus explaining its sensitivity to LV geometry. We further showed that a simple adjustment to compute EF using the mid-wall layer rather than the endocardial boundary removes this disadvantage [similar to mid-wall shortening (7)], enabling EF to be geometrically independent, and allowing it to provide unbiased comparisons between LV of different geometric shapes. The mid-wall EF is also more representative of global myocardial strain function (as indicated by GLS) than the traditional EF, and it is capable of distinguishing between HFpEF and healthy LVs where the traditional EF cannot (8, 9). Our previous work provided theoretical basis for the advantages of mid-wall EF over the traditional endocardial EF in cardiac function evaluation (6). However, the mid-wall EF has not been tested for its diagnostic and prognostic value.

Here, we propose a new EF parameter, adjusted EF (EFa) that can easily be obtained by routine echo scans, in replacement of EF. EFa effectively shifts the quantification of EF from the endocardial surface to the mid-wall layer, to resolve the above shortcomings of EF. It is more in line with functional indicators such as GLS than EF, which can conflict with functional indicators in scenarios such as HFpEF. In a large patient cohort, we show that EFa can provide additional diagnostic and prognostic information compared with traditional EF, particularly when EF is in the normal range.

## Method

### Adjusted ejection fraction (EFa)

Our proposed adjusted EF or EFa is obtained by multiplying a correction factor to the traditional endocardial EF ($EF_{\mathrm{endo}}$), via equations 1–2. This factor consists of a corrective term, α, that shifts the quantification of EF from the endocardial surface to the mid-wall layer, via an estimate of the LV mass (LVM), and a scaling term, 1.9, which scales the magnitude of EFa to be in a similar range as traditional EF, as informed by our patient data.


(1) $$\mathrm{EFa}=EF_{\mathrm{endo}}\;\times 1.9\alpha$$

(2) $$\alpha =\left(\frac{\mathrm{EDV}}{\mathrm{EDV}+\;0.5\times \mathrm{LVM}/\rho }\right)$$

Here, EDV is the LV end-diastolic volume, and ρ is the density of myocardium or 1.04 g/ml (10). The EDV and EF are calculated using modified Teichholz formulation. To estimate LVM, we used the Devereux formulae (calculated from m-mode) to estimate myocardial volume (11),

(3) $$\begin{array}{rl} & \mathrm{LVM}=\rho \times \mathrm{Myocardial}\;\mathrm{Volume}\\ & =1.04\{0.8\;\times \;[{\left(\mathrm{LVIDD}+\mathrm{PWT}+\mathrm{IVST}\right)}^{3}-\mathrm{LVID}D^{3}]+0.6\} \end{array}$$

where LVIDD is the LV inner diameter, PWT is the LV posterior wall thickness, and IVST is the inter-ventricular septal thickness, all evaluated at end-diastole. This formulation enables EFa to be easily calculated from a standard cardiac echo or MRI scan (6).

### Study design and patient population

We conducted a retrospective cohort study of patients admitted to Imperial College Healthcare NHS Trust (three-centre cohort), UK, between 2010 and 2017. Patients who had a request of a troponin test were enlisted in the study. Patients were included if they underwent an echocardiogram as part of their clinical assessment either within 3 months before or within 5 months after their admission to hospital, which may differ from their time of enlistment for study. There is no limit to the time between enlisting for study and their echocardiogram.

### Data source

This study involved the secondary use of existing data sources and did not include patients as study participants (12, 13). The data specification and acquisition process was registered at ClinicalTrials.gov, NCT03507309. Structured data from primary clinical systems were extracted in accordance with the Trusts clinical guidelines and subject to local quality and governance procedures. A data specification of variables was developed grouped into demographics, inpatient episodes, blood test results, diagnoses, cardiovascular procedures, echocardiography, and survival status. Patients who were admitted to the hospital had the International Classification of Diseases (ICD) discharge codes (14).

### Outcome

The main outcome of interest was risk of heart failure (HF) admission. Patients were classified as having a HF admission based on assigned ICD-10 codes (Table 1) and further classified into HFrEF, HFmrEF, and HFpEF based on their echocardiographic EF. All-cause mortality was assessed as a competing risk to HF admission. Life status was ascertained using routinely collected data on the NHS Spine application, which is linked to the Office for National Statistics and thereby to the national registry of deaths. The HF hospitalization data is tracked solely within the three centers and based on the discharging ICD codes.

**Table 1:** Baseline characteristics of patients according to LVEF.

		EF < 40 (*n* = 402)	40 ≤ EF < 50 (*n* = 293)	EF ≥ 50 (*n* = 2,057)	Total (*n* = 2,752)
Male, *n* (%)		65.9%	68.3%	53.0%	56.5%
Admitted due to: (when Echocardiogram is taken)	ACS, *n* (%)	45 (11.2%)	56 (19.1%)	211 (10.3%)	312 (11.3%)
	HF, *n* (%)	99 (24.6%)	34 (11.6%)	68 (3.3%)	201 (7.3%)
	Other causes, *n* (%)	258 (64.2%)	203 (69.3%)	1,778 (86.4%)	2,239 (81.4%)
History of:	HF,	13 (3.2%)	8 (2.7%)	12 (0.6%)	33 (1.2%)
*n* (%)	Previous MI, *n* (%)	107 (26.6%)	87 (29.7%)	282 (13.7%)	476 (17.3%)
	Diabetes, *n* (%)	58 (14.4%)	40 (13.7%)	160 (7.8%)	258 (9.4%)
	Obstructive Lung Disease, *n* (%)	11 (2.74%)	8 (2.7%)	63 (3.06%)	82 (3%)
Blood tests:	Normalized Troponin, (ng/L)/(ng/L)	1.9 [3.1µ,1.5k] (0.0%)	1.8 [3.1µ, 1.6k] (0.0%)	1.9 [3.1µ, 0.59k] (0.0%)	1.9 [3.1µ,0.96k] (0.0%)
Median [95%CI]	Creatinine, µmol/L	90 [56 450] (<0.1%)	89 [54 320] (<0.1%)	78 [50 280] (<0.1%)	81 [51 320] (<0.1%)
(% missing data in group)	Total Cholesterol, mmol/L	4.0 [2.3,6.6] (23.9%)	4.1 [2.2,7.0] (27.0%)	4.3 [2.3,7.1] (33.9%)	4.2 [2.3,7.0] (31.7%)
	CRP, mg/L	11 [0.6,240] (0.7%)	12 [0.6,230] (3.1%)	8.2 [0.4,260] (5.0%)	9.1 [0.4,260] (4.2%)
	eGFR, ml/min/1.73 m^2^	66 [12,91] (<0.1%)	65 [16,91] (<0.1%)	76 [17,91] (<0.1%)	74 [15,91] (<0.1%)
	Glucose, mmol/L	6.4 [3.8,19] (30.4%)	6.4 [4.2,25] (36.5%)	5.9 [4.0,22] (38.1%)	6.1 [4.0,22] (36.8%)
	Haemoglobin, g/dl	13 [8.5,130] (<0.1%)	13 [8.0,120] (<0.1%)	13 [8.5,130] (0.2%)	13 [8.5,130] (0.1%)
	HbA1c, mmol/mol	43 [31,100] (54.4%)	44 [30,98] (45.7%)	41 [31,98] (57.6%)	42 [30,99] (55.9%)
	HDL Cholesterol, mmol/L	1.1 [0.48,1.9] (25.4%)	1.1 [0.59,2.1] (29.0%)	1.1 [0.46,2.1] (35.7%)	1.1 [0.46,2.1] (33.5%)
	Platelet Count, 10^9^/L	220 [110,430] (<0.1%)	210 [110,450] (<0.1%)	230 [98,460] (0.2%)	230 [99,450] (0.1%)
	Potassium, mmol/L	4.3 [3.1,5.7] (<0.1%)	4.3 [3.3,5.8] (<0.1%)	4.2 [3.2,5.4] (0.4%)	4.2 [3.2,5.5] (0.3%)
	Sodium, mmol/L	138 [127,145] (<0.1%)	138 [126,144] (<0.1%)	138 [128,145] (<0.1%)	138 [127,145] (<0.1%)
	Triglycerides, mmol/L	1.1 [0.61,3.5] (24.2%)	1.2 [0.56,3.1] (27.0%)	1.3 [0.52,4.0] (34.0%)	1.2 [0.53,3.9] (31.8%)
	Urea, mmol/L	7.2 [3.2,25] (<0.1%)	7.0 [2.9,26] (<0.1%)	5.9 [2.4,22] (<0.1%)	6.2 [2.5,23] (<0.1%)
	White blood cell count, 10^9^/L	9.4 [4.3,20] (<0.1%)	9.0 [4.3,20] (<0.1%)	8.6 [4.0,20] (0.2%)	8.7 [4.1,20] (0.1%)
Rate of readmission with HF within 3 years, (% per year)		6.42	3.45	0.91	2.02

ACS, acute coronary syndrome; HF, heart failure; MI, myocardial infarction.

### Statistical analysis

This study was approved by the London South East Research Ethics Committee (REC reference: 16/HRA/3327).

The relationship between EF and EFa was evaluated using ordinary cubic least squares model. Patients were stratified into three groups based on their EF; (i) EF < 40, (ii) 40 ≤ EF < 50, and (iii) EF ≥ 50 (See Table 1). We investigate the diagnostic value of EFa by further stratifying the patients with EF ≥ 50 via their ICD-10 codes on the admission closest to their echocardiography to separate HFpEF patients and non-HF patients. In the analysis we used the adjusted *p* values of an ANOVA test to show significance difference between EFa and EF stratified by the groups mentioned above, as well as between non-HF patients and HF patients stratified by EF. We have also further stratified the HFpEF group into grades I, II and II according to ASE/EACVI guidelines (15) to show the difference in values of EF and EFa among the groups.

We investigated the long-term prognostic value of EFa on the risk of hospital readmission due to HF within 3 years. We first constructed a “baseline” multivariable Cox proportional hazards regression model that included age, sex and blood creatinine concentration (16) to associate with heart failure readmission. We then evaluated the effect of adding EFa, EF or LVM to this baseline model. Similar analyses were performed in the EF subgroups. The three model comparison metric gives a complete picture for switching from the baseline model to the new model: (i) (Integrated Discrimination Index) IDI represents the change in the accuracy of the overall risk score, (ii) Net Reclassification Index (NRI) indicates the proportion of patients who are more accurately assessed for risks of readmissions, and (iii) Akaike information criterion (AIC) and Bayesian information criterion (BIC) are estimators of error and model quality. The equations used are shown as Supplementary Equations 1–6. The analysis is repeated with a “15BT” model replacing the baseline model. The “15BT” model includes all the 15 blood test results listed in Table 1 with missing values (percentage missing is shown in Table 1) replaced with the population median. A leave one out cross validation is used to validate the models due to the low event count and presented in the receiver operating characteristic (ROC) curve analysis.

Due to the high mortality rate, we treated all-cause mortality as a competing event with HF readmissions and used the Fine and Gray model (17) to make necessary adjustments to the readmission risk before regression modelling (Supplementary Figure S1). This adjustment was used for all analyses calculating risk.

## Results

2,752 patients were admitted to the Imperial College Healthcare NHS Trust with the requests of a troponin test and had an echocardiogram performed within the inclusion criteria. Just over half of patients (56.5%) were men, mean age was 69.3 ± 16.4 years, and 312 (11.3%) were diagnosed with an ACS during their admission. The baseline characteristics of all patients and in the EF subgroups are shown in Table 1.

### Relationship between EFa and EF

**Figure 1:**
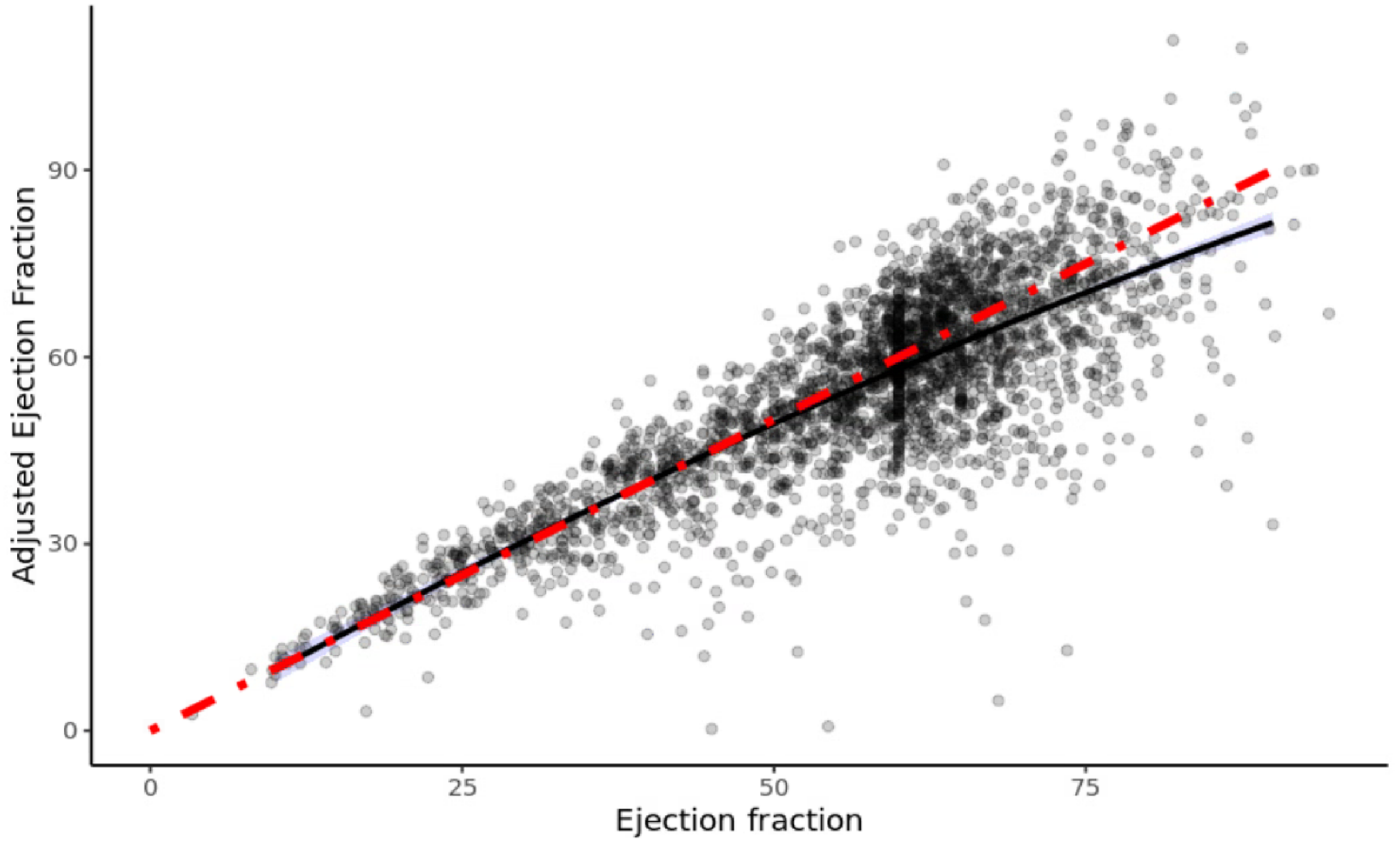
Efa versus EF of all patients in study. The red line denotes a 1:1 gradient relationship, while the black line denotes the least-square linear tail-restricted cubic spline regression line (overlapped with quadratic polynomial).

Figure 1 shows the plot of EFa vs. traditional EF for all patients, and the cubic least-square best fit relationship (black line) between the two. The magnitude of EFa is shown here to be very similar to that of traditional EF at low EF values, as demonstrated by the red line in Figure 1, which is the 1:1 gradient line. However, for EF ≥ 50, average EFa is found to be lower than the 1:1 relationship, suggesting that within the EF ≥ 50 cohort, EFa provides a different assessment of cardiac function from EF. The scaling term value of 1.9 is then used to scale the EFa to be in similar and clinically familiar range as the EF.

### Diagnosis with EFa

**Figure 2:**
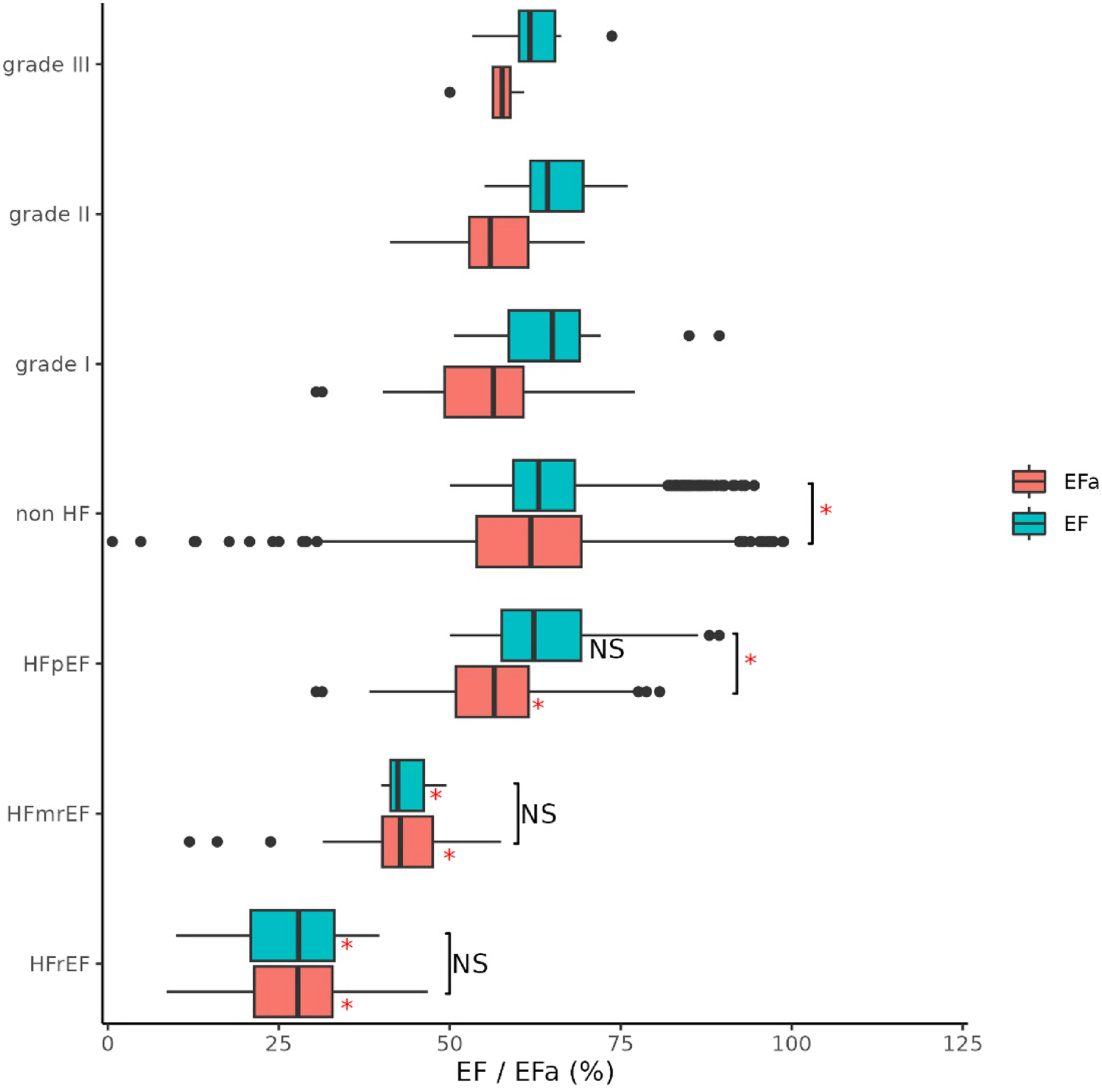
Ef and EFa of all patients, stratified into HFrEF (EF < 40), HFmrEF (40 ≤ EF < 50) and HFpEF (EF ≥ 50) and non-HF (not diagnosed with HF) based on ICD-10 codes. Notation “*” and “NS” refers to significant (adjusted *p* value < 0.05) and non-significant difference between respective groups (indicated with bracket) and with respective non-HF group (right of box) compared using ANOVA. LV diastolic dysfunction in HFpEF group as graded to I (*n* = 27), II (*n* = 27) and III (*n* = 7) according to ASE/EACVI guidelines.

Figure 2 shows the EF and EFa values in patients stratified into a non-HF cohort and cohorts with HFpEF (EF ≥ 50%), HFmrEF (40% ≤ EF < 50%), and HF with a reduced EF (HFrEF, EF < 40%) based on ICD-10 codes. The non-HF cohort are patients with EF ≥ 50% who did not have a diagnosis of HF at the time when these echocardiography measurements were conducted. Some of these patients were subsequently admitted due to HF. Here, we observe that EFa provides similar values to EF for the HFmrEF and HFrEF groups. Both EF and EFa can differentiate these groups from the non-HF group, which can be taken as a surrogate for a more normal group. However, EF is unable to discriminate between the HFpEF and non-HF group. In contrast, EFa is significantly different between the two groups. This corroborates our earlier animal studies (6), and emphasises the added value that EFa can provide in patients heart failure and a preserved ejection fraction. The HFpEF group is further stratified according to diastolic dysfunction grades. However, the number of patients in the HFpEF group which can be graded with available data is low and statistical significance cannot be established.

### Risk association with EFa

Table 2 shows resulting *p*-value of the change to risk association when EF, EFa, or LVM is added to the “Baseline” model. Here, the null-hypothesis is that there is no improvement to risk association, and thus a low *p*-value indicates a significant improvement to risk association.

**Table 2:** *P* value of hypothesis testing using IDI, NRI, AIC and BIC.

(a)	EF				EFa				LVM			
	EF ≥ 50	40 ≤ EF < 50	EF < 40	All patients	EF ≥ 50	40 ≤ EF < 50	EF < 40	All patients	EF ≥ 50	40 ≤ EF < 50	EF < 40	All patients
IDI	0.134	0.212	0.236	<0.001	0.003	0.072	0.055	<0.001	0.002	0.110	0.230	0.476
NRI	0.434	0.378	0.482	<0.001	0.006	0.133	0.238	<0.001	0.038	0.368	0.324	0.150
AIC, BIC	0.186	0.150	0.342	0.410	0.012	0.117	0.082	<0.001	0.058	0.002	0.086	0.002
(b)												
IDI	0.266	0.270	0.286	<0.001	0.050	0.252	0.244	<0.001				
NRI	0.288	0.207	0.107	<0.001	0.018	0.207	0.115	<0.001				

The null hypothesis is that risk association does not increase when either EF or EFa or LVM is added to the (a) “Baseline” model and (b) “15BT” model, for readmission risks (due to HF) within 3 years. The “Baseline” model utilizes age, sex and blood creatinine as confounders while the “15BT” model also includes other blood tests listed in Table 1. For AIC and BIC, only the higher *p* value was stated. Green highlight: *p* < 0.05 of lower or equal IDI/NRI, and high or equal AIC and BIC. See Supplementary Table 1 for shorter survival times.

Results for EF show that significance is generally obtained when analysis is applied to all patients, but not when it is applied to individual patient groups stratified by EF values. This confirms that EF broadly introduces an associative capability for readmissions due to HF to the baseline model, suggesting a prognostic value. However, within each group with stratified EF, no significance is observed, indicating that EF cannot provide associative capabilities beyond the baseline model within stratified groups. This is to be expected as EF is already used for the stratification. However, this implies that for the EF ≥ 50% group, which contains many HFpEF patients, EF does not associate with their readmission risks.

Table 2 also shows that when EFa is added to the baseline model, significance is observed for the analysis on all patients, indicating that EFa brings a broad improvement to associative capabilities similar to that brought by EF, and suggesting that EFa has a similar broad prognostic value as EF. On top of this, the *p* values for the EF ≥ 50% group is significant for all analyses. This suggests that even after the stratification with EF, a further evaluation with EFa can further improve HF readmission risk association for the EF ≥ 50% cohort, demonstrating that EFa has a novel, additional associative capability above that of EF. The repeated analysis with 15BT model shows that adding EFa into the model still improves the model in the EF ≥ 50 group, although less prominently, while the effect of adding EF remains the same.

### Improved prognosis with EFa compared to EF in the EF ≥ 50% cohort

**Figure 3:**
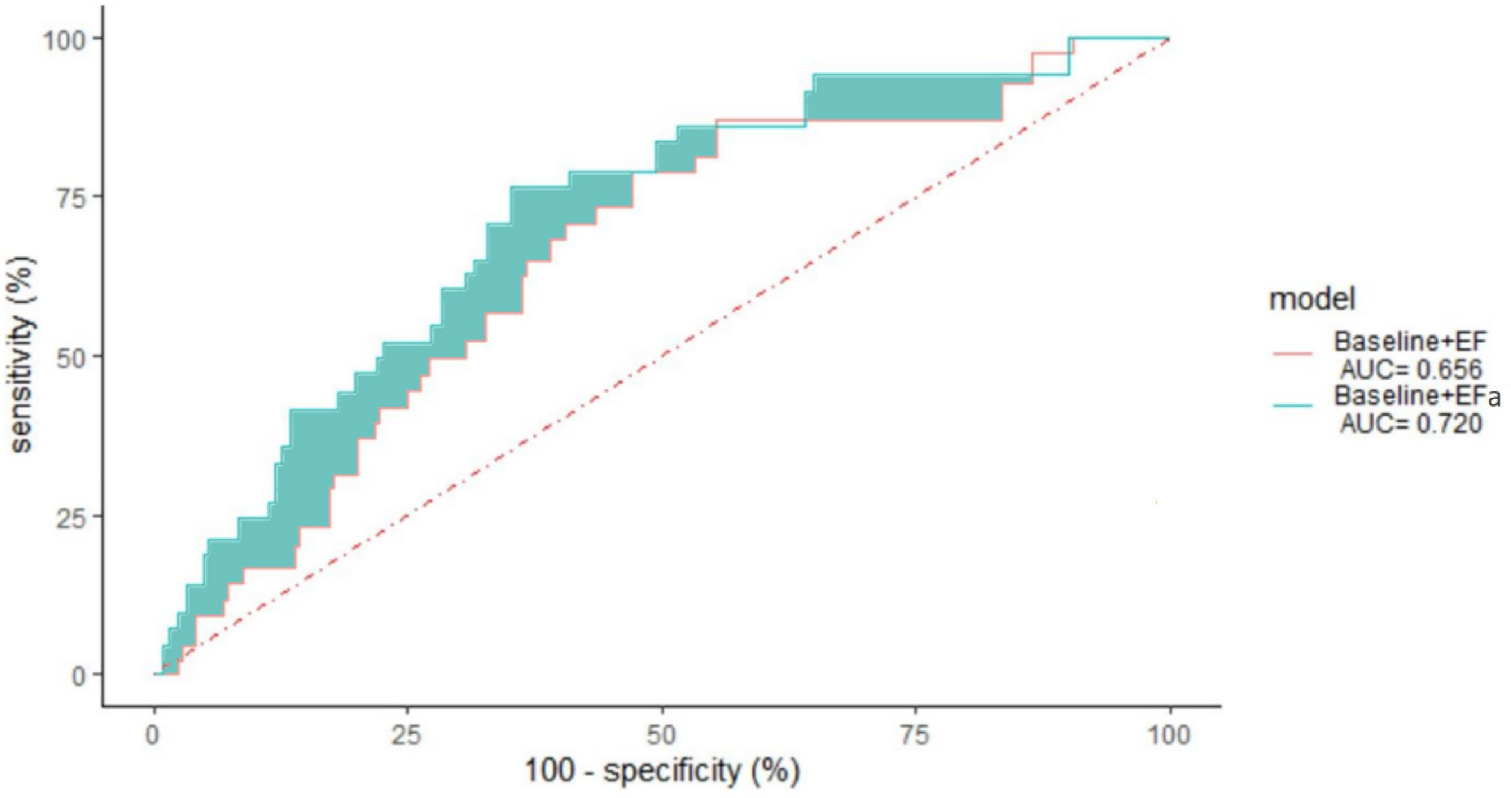
3 years non-admission ROC curve for patients with ejection fraction ≥ 50 using a leave one out cross validation of various models, “Baseline” with EF and “Baseline” with EFa. The area under the curve (AUC) is given in the legend. *p*-value = 0.007.

Next, we studied the long-term risk for readmissions due to HF (within 3 years) in the EF ≥ 50% cohort. Figure 3 shows the ROC curve for readmissions due to HF within 3 years, demonstrating a 18.6% reduction in error (quantified by area above the curve, AAC) when EFa replaces EF in the baseline risk models.

Table 3 shows the specific IDI and NRI scores to quantify the improvements to risk associations when EF or EFa are added to the baseline model, for assessing readmissions within 3 years in the EF ≥ 50% cohort. Here, the higher IDI and NRI scores for EFa compared to EF shows that adding EFa to “baseline” improves risk associations more than adding EF to “baseline”. The results also show that using EFa instead of EF will lead to 12.2% more patients in the event group (readmitted) being correctly identified as higher risk, and 16.6% more patients in the non-event group (not readmitted) being correctly identified as lower risk.

**Table 3:** 3-years readmissions NRI and IDI values of patients with EF ≥ 50, when comparing baseline model to baseline + EF model, comparing baseline model to baseline + EFa model, comparing 15BT model to 15BT + EF model and when comparing 15BT model to 15BT + EFa model.

Patients with EF ≥ 50, assessment for readmissions within 3 years	Baseline + EF	Baseline + EFa	15BT + EF	15BT + EFa
IDI (×10^−3^)	1.168	6.435	−0.580	5.216
event risk score changes (×10^−3^)	1.398	6.026	0.027	5.458
non event risk score changes (×10^−3^)	0.230	−0.408	0.607	0.242
NRI	0.065	0.352	0.113	0.297
proportion of patient with increased risk score in event group (>50% indicates improvement)	58.4%	70.6%	63.7%	68.1%
proportion of patient with decreased risk score in non-event group (>50% indicates improvement)	48.1%	64.7%	47.6%	61.6%

**Figure 4:**
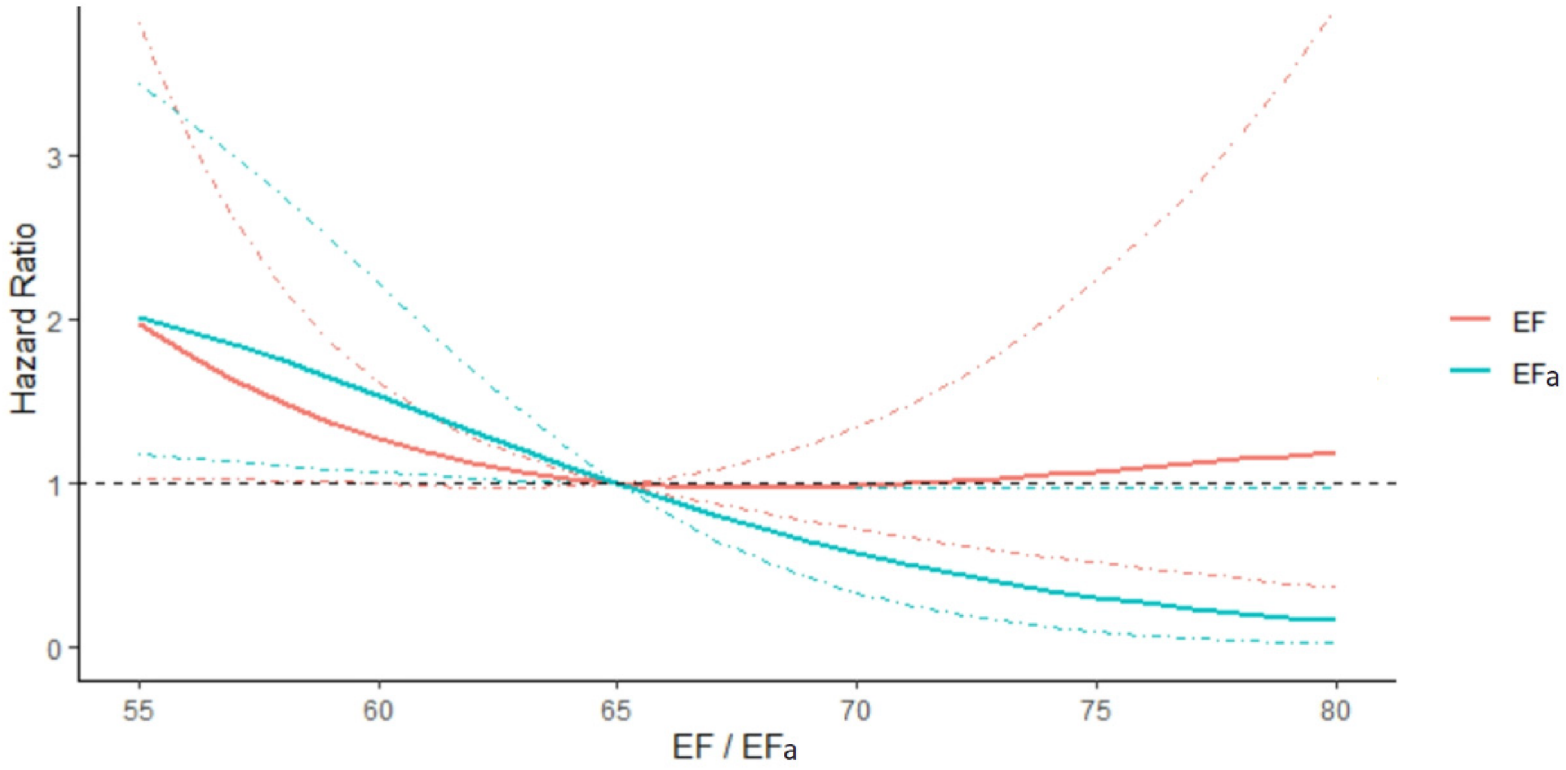
Hazard ratio of readmissions due to HF within 3 years, plotted against EF and EFa for patients with EF ≥ 50. The hazards are compared to that for EF or EFa is 65. The models are adjusted for the effects of age, sex, and blood creatinine concentration. 95% Confidence intervals are shown in dotted lines of the corresponding colour.

Figure 4 shows the plot of hazard ratio of readmissions due to HF within 3 years, plotted against EF or EFa, for the cohort where EF ≥ 50%. Here, we observe that the hazard ratio does not change with increasing EF for a substantial range of high EF, suggesting a poor ability of EF to indicate risks of readmissions due to HF in this cohort. In comparison, the hazard ratio shows a clear trend of decreasing when EFa increases, showing EFa is more successful at indicating HF readmission risk. Similar results are obtained for readmissions over other follow up periods (Supplementary Figure S2).

### Role of formulation and LVM in EFa's associative capabilities

**Figure 5:**
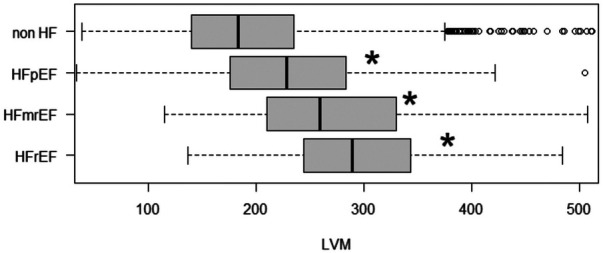
LVM of patients admitted due to HF (grouped into HFrEF, HFmrEF and HFpEF) and non HF admissions. HF are grouped into HFrEF (EF < 40), HFmrEF (40 ≤ EF < 50) and HFpEF (EF ≥ 50). Notation “*” and “NS” refers to significant (adjusted *p* value < 0.05) difference between respective groups compared with the non HF group using ANOVA.

The EFa is in essence an adjustment to traditional EF using LVM, in a theoretical way that is equivalent to quantifying EF at the mid-wall location (6). Here, we test the hypothesis that the improved diagnosis and prognosis capabilities of EFa over EF is due to this additional consideration of LVM, which allows better stratification of HFpEF cases. Figure 5 demonstrates that all HF groups have significantly increased LVM compared to the non-HF cohort, highlighting the importance of considerations of LVM. Adding LVM to the baseline model (Table 2) improved its associative capability for long-term HF readmissions in the EF ≥ 50% group. The effects of this addition of LVM to “baseline” is shown in Table 4, which shows that IDI and NRI increases, indicating higher risk association, and the risk score accuracy improves. This is very similar to the effects of adding EFa to “baseline”. These evidence supports the hypothesis that EFa incorporates the risk association of LVM into the EF measurement.

**Table 4:** 3-years readmissions NRI and IDI values of patients with EF ≥ 50, when comparing baseline model to baseline + EFa model, and when comparing baseline model to baseline + LVM model.

Patients with EF ≥ 50, assessment for readmissions within 3 years	Baseline + EFa	Baseline + LVM
IDI (×10^−3^)	6.435	6.758
event risk score changes (×10^−3^)	6.026	7.46
non event risk score changes (×10^−3^)	−0.408	0.701
NRI	0.352	0.163
proportion of patient with increased risk score in event group (>50% indicates improvement)	70.6%	49.6%
proportion of patient with decreased risk score in non-event group (>50% indicates improvement)	64.7%	66.7%

However, we found that the simple inclusion of LVM in the model does not perform as well as using EFa. Firstly, ROC analysis for readmissions due to HF within 3 years shows that using EFa + the “baseline” model outperforms using LVM + the baseline” model with the error (quantified as area above the curve) being reduced by 4.4% (Supplementary Figure S3). Secondly, when we evaluated the effects of adding EFa to the EF + LVM + the baseline model (Supplementary Tables S2, S3), we find that adding EFa still brings about an improvement in the risk association for cases where the EF is high.

## Discussion

In the current study, we demonstrate that a proposed correction for EF using LVM improves the diagnostic and prognostic capabilities of EF. This correction factor is formulated with a theoretical basis that is equivalent to calculating EF at the mid-wall location rather than the endocardial location (6), which we previously showed can bring two advantages. Firstly, it enables EF to be more representative of the global strain function of the heart, and thus act as a confirmation of functional assessments via strains to complement strain measurements. Secondly, it resolves the shortcoming of EF, where geometric changes to the LV, such as from cardiac size remodelling, can alter the magnitude of EF, even if there is no change to the flow or strain function of the heart (6). We do not propose EFa to be a replacement of GLS and other functional parameters, but for it to completement these other parameters, and to ensure the EF measure will not conflict with functional parameters (such as EF indicating normality while functional parameters indicate disease).

There is much literature that corroborates our proposal that such a correction can bring advantages. Firstly, myocardial strain and fractional shortening (FS) measurements, which are typically done at the mid-wall layer, have shown good diagnostic capabilities, and have been found to be good indicators of heart failure (18–20). Secondly, past study comparisons between mid-wall FS and endocardial FS revealed that endocardial FS cannot identify reduced LV function when concentric hypertrophic occurs (21). Further, mid-wall FS has been found to have prognostic value, with low mid-wall FS being correlated with a higher likelihood for future morbid event (22).

The adjusted EF (EFa) is a suitable replacement for EF, because it can properly discriminate HFpEF patients from healthy hearts when in cases with high EF, but at the same time, in cases with lower EF, where EF demonstrates strong clinical utility and is frequently used, EF and EFa are hardly different. However, despite more robust diagnostic and prognosis abilities, we do not expect EFa to replace more advanced metrics such as GLS, mid-wall FS and mid-wall EF in detailed diagnosis processes, as EFa is an estimate of these strain and deformation functional parameters, and as such will not do better than these parameters. EFa's main utility is to avoid the need to use a parameter that conflicts with functional parameters, the traditional EF.

By comparing various models, we show that the EFa enhances risk association in the cohort of patients where EF ≥ 50% when it is added to the model. We also demonstrate this with a model including blood test covariates (the 15BT model). We further demonstrate evidence that this associative ability is enhanced due to the inclusion of LVM and this new model is able to differentiate between non-HF patients and HFpEF patients, which is something that traditional EF cannot do. Our data suggest that there is added value in evaluating EFa after clinical stratification with EF in those patients with an apparently normal EF. Doing this separates these patients further and may identify a further group of patients who may derive prognostic benefit from some heart failure medications that have been shown to benefit those with HFrEF. Cases with high EF but low EFa can be flagged for closer evaluation. Previous drug interventional studies on patients with HFpEF may be reanalysed with this methodology to if there is any evidence to support the group of patients with a lower EFa deriving prognostic benefit from traditional heart failure medication.

Importantly, the calculation of EFa in patients with an EF of 40% or less, results in very similar numbers to the EF calculation. Therefore, EFa retains the ability to detect patients who have been shown to benefit from prognosis improving treatments in randomised controlled trials.

We found that adding EFa to a model which already have EF and LVM improves the model further. We propose that this is because EFa has a good theoretical formation (6), and its inclusion of LVM is via a mathematical formation that ties the adjusted EF measure more closely to function, which cannot be achieved by simple inclusion of LVM into the model.

In our current approach, the EFa is calculated with EF, LVM, and EDV, all of which are easily measured with routine echo scans, suggesting low technical and logistical barriers to adoption in echo routine. There are other approaches to obtain a mid-wall EF, such as tracing the location of the mid-wall layer and at systole and diastole (8, 9), which are likely to achieve similar good results, but such approaches require new measurement procedures, and can have errors if the tracing is done manually. The clear results obtained in this study suggest that despite its simplicity, this approach is effective.

### Study limitations

The inclusion of a patient is an echocardiogram performed with a past request of a troponin test which causes the study population to be heterogeneous compared to a HF study group. Thus the database includes many competing risk for all-cause mortality that is difficult to differentiate from HF mortality. Therefore, we used a softer end point that is more directly related to HF, which is HF readmission. To address the competing effect of all-cause mortality with HF readmission, we employed the Fine and Gray model, but acknowledge that this may not eliminate the competition completely. Further, as the database focuses on patients who were admitted with the request of a troponin test, there might be a lack of a control group who are free from underlying conditions.

Regarding the limitation of the end point, a standardized diagnostic process of the ICD codes is not defined in detail for all the clinical team to use and is potentially subjected to uneven diagnostic process. Moreover, we do not have NTproBNP/BNP results to verify the accuracy of the diagnosis. This also limits the capability of our Cox proportional hazards regression models. Further, distinction among specific myocardiopathies, comorbidities, and treatments were also not considered in this analysis as such data is incomplete and not analysable.

In terms of the calculation of EFa, the correction factor used is a simplified approach. The half myocardial volume approach is only an approximation for the mid-wall location, and a single M-mode measurement is used to obtain wall thickness for LVM quantification rather than multiple measurements across different segments. There can be more elaborate and accurate methods, such as quantifications with 3D scans, or measurements at multiple segments. Nonetheless, EFc is associated with HF admission risk despite such inaccuracies. More accurate quantification of LV and myocardial volume may improve the association, but such approaches are likely logistically more challenging, and may discourage the adoption of EFa.

Lastly, correlation of EFa with standard mid-wall EF is not examined due to the difficulty to consistently obtain standard mid-wall EF automatically for large database and the relevance of the correlation in the associating with HF admission risk.

## Conclusion

We propose a correction factor for EF that inserts considerations for LVM. This enhances the ability of EF to categorise patients with HFpEF better, which may lead to improved treatments for these patients. This novel measurement, EFa, improves the association with hospital admissions due to HF for patients where the EF ≥ 50%.

## Data Availability

The original contributions presented in the study are included in the article/**Supplementary Material**, further inquiries can be directed to the corresponding author.
