# Supplementary material for: Diagnostic and prognostic value of an ejection fraction adjusted for myocardial remodeling
Source: Front Cardiovasc Med. 2024 May 9;11:1349338. doi: 10.3389/fcvm.2024.1349338 (PMC11122018; doi:10.3389/fcvm.2024.1349338)
Supplement: Supplementary file 1 [file Datasheet1.docx]

# Supplementary Material

# **Derivation of EFa**

Our adjusted EF (EFa) is calculated by converting EF’s evaluation from the endocardial surface to the mid-wall location by applying a factor, $\alpha$ to EF, followed by applying another factor of 1.9 to ensure EFa is of the same order of magnitude as EF in normal persons. Its derivation is as follows. First, we noted that the traditional EF is expressed as,

$$EF=\frac{EDV_{endo}-ESV_{endo}}{EDV_{endo}}$$

Where EDV is end diastolic volume, and ESV is end diastolic volume, and the subscript endo signifies that these volumes are calculated at the endocardial surface. To shift these volume measures from the endocardial surface to the mid-wall location (denoted with the “mid-wall” subscript), we simply need to add half of the myocardium volume ($Vol_{LV}$) to them:

$$E{SV}_{mid-wall}=ESV_{endo}+0.5\times Vol_{LV}$$

$$E{DV}_{mid-wall}=EDV_{endo}+0.5\times Vol_{LV}$$

We can then express EFa using these mid-wall volume measures:

$$EFa=\frac{EDV_{mid-wall}-ESV_{mid-wall}}{EDV_{mid-wall}}$$

$$EFa=\frac{\left( EDV_{endo}+0.5\times Vol_{LV} \right)-(ESV_{endo}+0.5\times Vol_{LV})}{EDV_{endo}+0.5\times Vol_{LV}}$$

$$EFa=\frac{EDV_{endo}-ESV_{endo}}{EDV_{endo}} \left( \frac{EDV_{endo}}{EDV_{endo}+0.5\times Vol_{LV}} \right)$$

$$EFa=EF\times\alpha$$

Where

$$\alpha=\frac{EDV_{endo}}{EDV_{endo}+0.5\times Vol_{LV}}=\frac{EDV_{endo}}{EDV_{endo}+0.5\times LVM/\rho}$$

Next, we add a 1.9 factor which is empirically obtained, so that EFa will be in the same order of magnitude as EF:

$$EFa=EF\times1.9\alpha$$

It is noteworthy that our approach of calculating EFa does not achieve the calculation of EF at the mid-wall location in the strict definition of mid-wall to be equidistant from the epicardial and endocardial locations, since the myocardial wall is curved and the volume inside and outside of this strict definition is not equal. Our approach is thus an estimate of the mid-wall location rather than the true mid-wall location.

# **Supplementary Results**

**
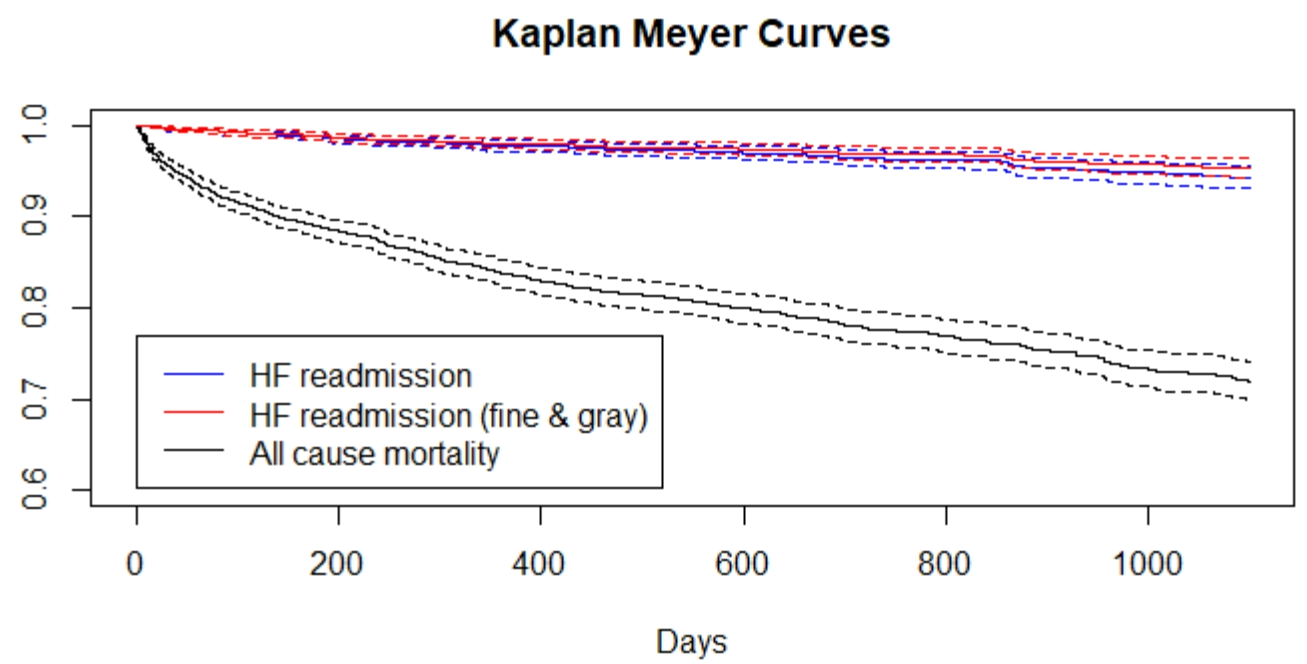
**

**Supplementary Figure 1.** **Kaplan-Meier curves for readmission due to HF and all-cause mortality as well the readmission due to HF adjusted for competing risk with the Fine and Gray model. Dotted line represents the 95% confidence interval.**

Supplementary Figure 2 shows the hazard ratio for readmissions within 90 days, 180 days, 1 year and 3 years, for various EFa values, after a correction for the effects of age, sex and blood creatinine concentration. The hazard ratio increased with reduced EFa for short-, mid-, and long-term. The confidence intervals are narrower for 1 and 3 years readmissions due to higher sample sizes.


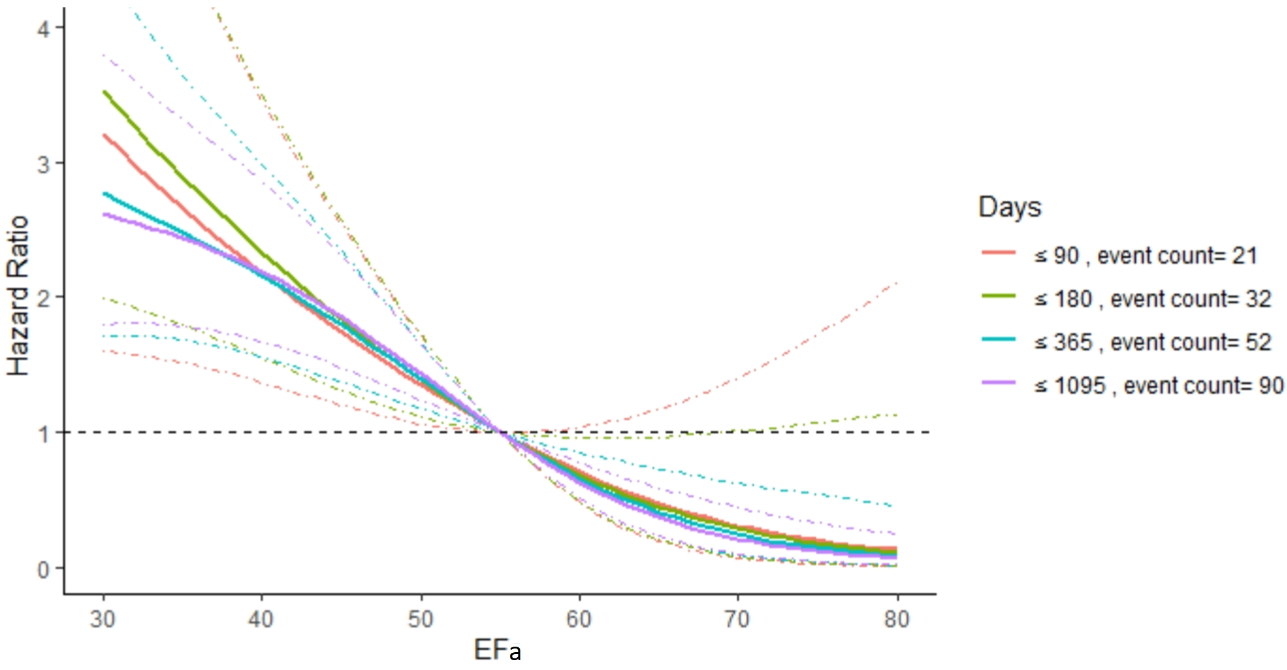


**Supplementary Figure 2. Short-, middle-, and long-term hazard ratio plotted versus EFa. The hazards are compared to the respective hazard time and EFa of 55 for HF readmission. The models are adjusted for age, sex and blood creatinine concentration. 95% Confidence intervals are shown in dotted lines of the corresponding color.**

Supplementary Table 1 shows the full results for hypothesis testing that the addition of EF or EFa improves risk association of the model, where testing is done for readmissions due to HF within various time durations.

| **Analysis Method** | **Duration** | **EF** | | | | **EFa** | | | |
| --- | --- | --- | --- | --- | --- | --- | --- | --- | --- |
|  |  | EF≥50 | 40≤EF<50 | EF<40 | All patients | EF≥50 | 40≤EF<50 | EF<40 | All patients |
| IDI | Day 90 | 0.186 | 0.320 | 0.308 | 0.002 | 0.047 | 0.157 | 0.096 | <0.001 |
|  | Day 180 | 0.134 | 0.332 | 0.424 | <0.001 | 0.060 | 0.145 | 0.168 | <0.001 |
|  | Day 365 | 0.180 | 0.500 | 0.440 | <0.001 | 0.008 | 0.114 | 0.180 | <0.001 |
|  | Day 1095 | 0.134 | 0.212 | 0.236 | <0.001 | 0.003 | 0.072 | 0.055 | <0.001 |
| NRI | Day 90 | 0.104 | 0.332 | 0.396 | <0.001 | 0.021 | 0.155 | 0.200 | <0.001 |
|  | Day 180 | 0.206 | 0.320 | 0.326 | <0.001 | 0.078 | 0.181 | 0.140 | <0.001 |
|  | Day 365 | 0.184 | 0.332 | 0.450 | <0.001 | 0.092 | 0.169 | 0.223 | <0.001 |
|  | Day 1095 | 0.434 | 0.378 | 0.482 | <0.001 | 0.006 | 0.133 | 0.238 | <0.001 |
| AIC,  BIC | Day 90 | 0.270 | 0.196 | 0.268 | <0.001 | 0.002 | 0.126 | 0.107 | <0.001 |
|  | Day 180 | 0.116 | 0.394 | 0.476 | <0.001 | 0.007 | 0.223 | 0.222 | <0.001 |
|  | Day 365 | 0.134 | 0.182 | 0.314 | 0.222 | 0.015 | 0.152 | 0.025 | 0.175 |
|  | Day 1095 | 0.186 | 0.150 | 0.342 | 0.410 | 0.012 | 0.117 | 0.082 | <0.001 |

**Supplementary Table 1. P value of hypothesis testing using IDI, NRI, AIC and BIC. The null hypothesis is that risk association of readmission due to HF does not increase when either EF or EFa is added to the “Baseline” mode. The Baseline utilizes age, sex and blood creatinine as confounders. Duration indicates the time duration within which readmissions occurs. For AIC and BIC, only the higher p value was stated. Green highlight: p<0.05.**

When we compared the EF + LVM + the baseline model to one where EFa is further added, we still observed significantly enhanced associative capabilities (Supplementary Table 2) for long-term (within 3 years) readmissions due to HF, in cohorts of patients with high EF (≥55% and ≥60%). This indicates that, although EFa is essentially an inclusion of LVM to EF, simply including LVM and EF in the model does not associate with readmissions as well as including EFa. Supplementary Table 3 further shows the effects of using EFa rather than LVM and EF, showing modest improvement in positive associations in the event group and negative associations in the non-event group.


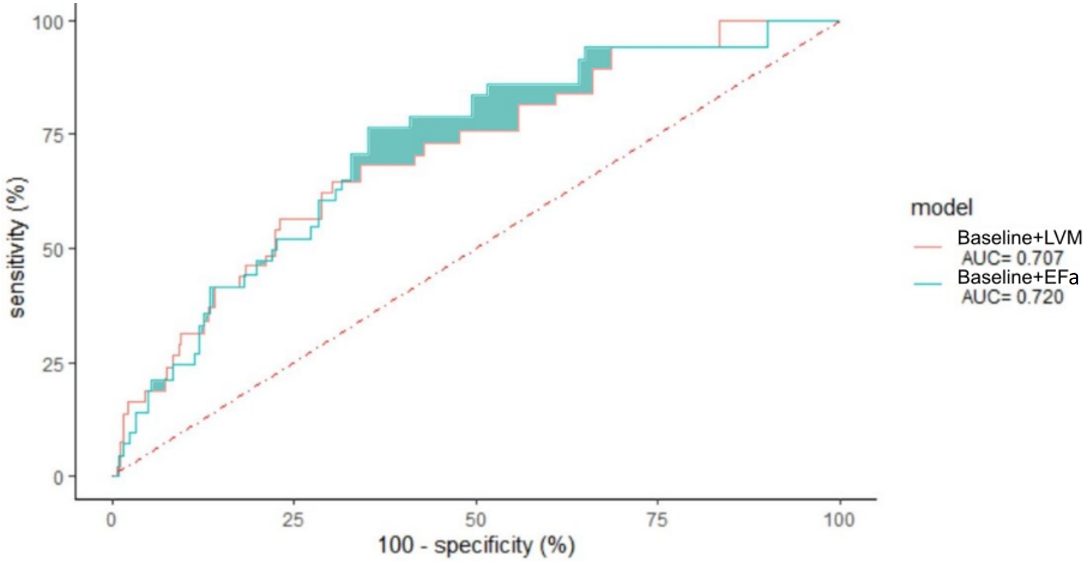


**Supplementary Figure 3. 3 years non-admission ROC curve for patients with ejection fraction ≥ 50 using a leave one out cross validation of various models, “Baseline” and “Baseline” with LVM. The area under the curve (AUC) is given in the legend. p-value = 0.30.**

| **Day 1095** | **EF≥50** | **EF≥55** | **EF≥60** | **All patients** |
| --- | --- | --- | --- | --- |
| IDI | 0.164 | 0.042 | 0.028 | 0.252 |
| NRI | 0.314 | 0.114 | 0.072 | 0.322 |
| AIC, BIC | 0.072 | 0.042 | 0.048 | 0.186 |

**Supplementary Table 2. P value of hypothesis testing using IDI, NRI, AIC and BIC. The null hypothesis is that risk association of readmission due to HF does not increase when EFa is added to an initial model. The initial utilizes EF, LVM, age, sex, blood creatinine as confounders. For AIC and BIC, only the higher p value was stated. Green highlight: p<0.05.**

| **1095 Days** | **IDI** (✕10^-3^) | **IDI**  **event risk score changes** (✕10^-3^) | **IDI**  **non event risk score changes** (✕10^-3^) | **NRI** | **NRI**  **proportion of patient with increased risk score in event group** | **NRI**  **proportion of patient with decreased risk score in non event group** |
| --- | --- | --- | --- | --- | --- | --- |
| **EF≥55** | 4.541 | 3.747 | -0.794 | 0.103 | 43.2% | 67.1% |
| **EF≥60** | 7.466 | 6.911 | -0.555 | 0.192 | 49.7% | 69.5% |

**Supplementary Table 3. 3-years survival NRI and IDI values of patients with EF≥55 and EF≥60, quantifying the improvements provided when EFa is added to the model that uses EF, LVM, age, sex, and blood creatinine concentration.**
